# OsLB2.2 negatively regulates rice disease resistance at seedling stage in rice

**DOI:** 10.3389/fpls.2025.1629283

**Published:** 2025-08-11

**Authors:** Tianze Ma, Mengge Wang, Shupeng Xie, Chengxin Li, Jingguo Wang, Hualong Liu, Luomiao Yang, Longnan Men, Zhonghua Sun, Tianpeng Zong, Detang Zou, Hongliang Zheng, Wei Xin

**Affiliations:** ^1^ Key Laboratory of Germplasm Enhancement and Physiology & Ecology of Food Crops in Cold Region, Ministry of Education, Northeast Agricultural University, Harbin, China; ^2^ Heilongjiang Academy of Agricultural Sciences, Suihua, China; ^3^ Harbin Academy of Agricultural Sciences, Harbin, China; ^4^ Tianjin Tianlong Science & Technology Company Limited, Tianjin, China

**Keywords:** rice, blast, genome-wide association study, RNA-seq, resistance gene

## Abstract

**Background:**

Rice blast disease, caused by the fungal pathogen *Magnaporthe oryzae*, stands as the most destructive diseaset of rice, negatively impacting yield and quality. Identification of blast resistance genes is crucial for breeding disease resistant varieties. In this study, we conducted a genome-wide association analysis on rice blast with 295 Japonica rice varieties and the rice blast dominant physiological race ZD5 from Heilongjiang.

**Results:**

A total of 11 Quantitative Trait Loci (QTLs) encompassing 233 genes were mapped. Notably, 40 genes showed significant phenotypic variations among different haplotypes. By combining GWAS and RNA sequencing analysis, we identified five candidate genes related to rice seedling resistance to rice blast. Through the assessment of relative gene expression levels in 10 susceptible and 10 resistant varieties, *OsLB2.2* (*LOC_Os02g57470*) emerged as a key gene displaying significant expression differences in resistant varieties under uninoculated and inoculated conditions. To elucidate gene functionality, we generated *oslb2.2* knockout mutants. The results showed that *oslb2.2* knockout mutants were significantly enhanced rice blast resistance compared to the wild type. Furthermore, we identified the dominant haplotype of *OsLB2.2* and developed Kompetitive Allele - Specific PCR (KASP) molecular markers for molecular improvement of rice blast resistance.

**Conclusion:**

The phenotypic validation indicated that OsLB2.2 negatively regulated the rice blast resistance at the seedling stage. Moreover, KASP molecular markers were developed, providing a theoretical basis for the potential application of OsLB2.2 in molecular breeding strategies.

## 1 Introduction

Rice (*Oryza sativa*) holds vital significance as a primary food crop, serving as the staple diet for a substantial portion of the global populace. The safe and sustainable cultivation of rice is crucial for upholding social stability and fostering economic progress (Elert, 2014). Rice blast (*Magnaporthe oryzae*), recognized as a severe rice disease on a worldwide scale, has been documented in more than 80 countries, resulting in annual yield losses nearing 30% (Guo et al., 2018; Zhang et al., 2022). Rice blast has emerged as a prominent factor constraining the stability and high productivity of rice cultivation (Zhang et al., 2019). Therefore, the prevention of rice blast stands as a pressing priority for rice production on a worldwide scale. Empirical evidence underscores that the cultivation of blast-resistant rice varieties represent the most cost-effective and efficient strategies for managing rice blast outbreaks (Wang et al., 2024).

To date, more than 100 rice blast resistance genes have been characterized, with 41 of them cloned (Wang et al., 2022a; Devanna et al., 2022). Among the cloned resistance genes, 37 are associated with nucleotide-binding site-leucine-rich repeat (NBS-LRR) proteins. The NBS domain is comprised of three highly conserved kinase motifs that facilitate phosphate binding to generate energy for pathogen defense mechanisms, while the C-terminal LRR region is involved in protein-protein interactions between resistance genes and *Avr* genes (Wang et al., 1999; McHale et al., 2006). The NBS-LRR gene is frequently arranged in tandem repeats to orchestrate rice blast resistance mechanisms (Dubey and Singh, 2018; Tanweer et al., 2015). *Piz-t*, *Pigm*, *Pi50*, *Pi9* and *Pi2* are clustered within the pericentric region of rice chromosome 6, sharing a common genetic locus and collectively contributing to enhancing rice resistance against blast pathogens (Wang et al., 2017). The Pik locus, located at the terminal end of the long arm of rice chromosome 11, encompasses multiple blast resistance *R* genes: *Pik*, *Pikm*, *Pikp*, *Piks*, *Pikh*, and *Pi1*, which collectively contribute to bolstering rice resistance against blast pathogens (Ying et al., 2022). In addition, *Pi-d2* (Chen et al., 2006), *pi21* (Fukuoka et al., 2009), *Ptr* (Zhao et al., 2018), and *bsr-k*1 (Tanweer et al., 2015) encode receptor-like kinases, proline-rich proteins, atypical proteins with four armadillo repeats, and proteins with tetratricopeptide repeats, respectively. Nevertheless, the rapid evolution of the pathogen *Magnaporthe oryzae* often leads to a decline in the resistance levels of previously resistant rice varieties after multiple years of widespread cultivation (Ni et al., 2019). Therefore, the exploration and identification of novel blast resistance genes for the breeding of new rice varieties resistant to rice blast hold significant importance.

Recently, large-scale approaches such as genome-wide association study (GWAS) and RNA sequencing (RNA-seq) have been employed to elucidate the molecular underpinnings of plant defense mechanisms. GWAS, a technology for genome-wide genetic marker detection, has emerged as a valuable tool for exploring the genetic basis of complex diseases (Moore et al., 2010). Compared with traditional parent-offspring linkage analysis, GWAS is highly efficient in detecting QTLs in natural plant populations. It does not require the construction of specific populations and can directly utilize natural populations, saving time and costs while covering more natural variations. At the same time, it has higher resolution and can locate to kb-level tiny genomic regions, while parent-offspring linkage analysis relies on specific hybrid populations, with the located intervals being several Mb to tens of Mb wide, and further fine mapping is needed (Yano et al., 2016; Bohra et al., 2020; Tibbs Cortes et al., 2021 (Yu et al., 2022)). In cotton, GWAS conducted on 335 U.S. Upland cotton accessions and utilizing 26,301 Single Nucleotide Polymorphic (SNP) markers identified 11 QTLs linked to resistance against *Xanthomonas citri* pv*. malvacearum* (Xcm) race 18 (Elassbli et al., 2021). In soybean, GWAS conducted on 350 germplasm resources and utilizing 52,041 high-quality SNP markers identified 8 SNP loci significantly linked to *Fusarium oxysporum* root rot resistance (Sang et al., 2023). In rice, a novel wall-associated kinase (*WAK*) gene, *Pb4*, conferring resistance to rice blast, was discovered through GWAS analysis involving 249 rice varieties (Fan et al., 2024). The RNA-seq has emerged as a potent technology for investigating the transcriptional interplay between host plants and pathogens (Wang et al., 2020; Pérez-Torres et al., 2021). For instance, transcriptome analysis of both resistant and susceptible maize plants under *Fusarium graminearum* infection led to the identification of 897 specific genes associated with *Gibberella* ear rot (Yuan et al., 2020). Similarly, an analysis of rice transcriptomes following infection by various rice blast races at different time points revealed 5 genes associated with rice blast resistance (Tao et al., 2021). The genetic underpinnings of numerous complex traits have been investigated by combining GWAS and RNA-seq analysis. In maize, two genes associated with low temperature tolerance during germination were identified (Zhang et al., 2020). Rice research uncovered two genes encoding NBS-LRR proteins that confer resistance to bacterial leaf blight (Shu et al., 2021).

In this study, a rice blast resistance-related gene, *OsLB2.2*, was identified by combining GWAS and RNA-seq analysis. The CRISPR/Cas9 gene editing knock-down expression mutant, *oslb2.2* (KO1, KO2), were used as experimental materials to carry out the current study. The function of *OsLB2.2* in rice blast resistance was analyzed, and the kompetitive allele specific PCR (KASP) molecular marker for *OsLB2.2* was developed. The results of this study can provide valuable genetic resources and germplasm resources for rice blast resistance breeding.

## 2 Materials and methods

### 2.1 Plant materials and growth conditions

GWAS was conducted using 295 diverse rice genotypes, which were collected from the Heilongjiang, Jilin and Liaoning provinces in China and other countries including Japan, the Republic of Korea, the Democratic People’s Republic of Korea and Russia. Rice seeds were soaked at 32°C for 2 days, then sown in plastic seedling trays (60 cm × 30 cm × 4 cm) filled with soil. Six seeds were placed in each hole of the trays, and set up for 7 replications. The seeded seedling trays were transferred to a growth chamber, with a cycle of 14 h light/26°C and 10 h darkness/24°C. Rice seedlings were cultivated until the third-to-fourth leaf stage for blast inoculation.

### 2.2 Blast pathogen and the inoculation procedure

The strain of the dominant physiological race ZD5 of rice blast in Heilongjiang was provided by the Rice Research Institute of Northeast Agricultural University. The rice blast strain was activated on potato medium for one week before undergoing propagation. It was cultured on potato medium at 26°C for 7 d, after which the aerial hyphae were removed, rinsed with sterile distilled water, dried, and covered with multilayer gauze. The culture was then subjected to a 12 h light and 12 h dark cycle for 7 d to stimulate spore production. Spores were washed with a sterile 0.10% Tween-20 solution. The wash suspension was transferred to sterile centrifuge tubes and centrifuged at 3000 rpm for 5 min. After carefully discarding the supernatant, the pellet was resuspended in an appropriate volume of 0.10% Tween-20 solution. Spore concentration was determined by hemocytometer counting under an optical microscope with three independent replicates, and the mean value was calculated. Finally, the spore suspension was precisely adjusted to 1.0×10^5^/mL using 0.10% Tween-20 solution. Following spray inoculation, the samples were placed under constant temperature conditions at 28°C with over 95% relative humidity in the dark for 24 h to ensure thorough fungal infection of the tested varieties, thereby facilitating disease progression. The disease status was evaluated 7 days after inoculation. The leaf diseases were scored according to the 0-9 grading standard of the International Rice Research Institute (IRRI) standard evaluation system (SES). (International Rice Research Institute [IRRI], 2014) (**Supplementary Table S1**).

### 2.3 Genome-wide association studies

Plink 2.0 software (Chang et al., 2015) was used to screen 788,396 SNPs developed by re-sequencing of 295 rice varieties (minor allele frequency (MAF) > 5%, missing rate < 20%) (Li et al., 2019b). GWAS was performed using the mixed linear model (MLM) in TASSEL 5.0 (Bradbury et al., 2007), with population structure Q-values from ADMIXTURE analysis as covariates and the kinship matrix (K) as a random effect, utilizing population SNP genotype data to identify trait-marker associations and determine significant loci. The threshold for the identification of SNPs significantly associated with traits was set to P < 5.46 × 10− 6, determined by genetic type 1 error calculator (GEC; http://statgenpro.psychiatry.hku.hk/gec/), which calculates the effective number of independent markers. The Manhattan plot was generated using the CMplot package of R (Yin, 2020). Based on previous studies conducted in our laboratory (Li et al., 2019b), the average linkage disequilibrium (LD) value within this natural population is 109 kb. To determine significant sites, if two or more significant SNPs were located in the same LD interval, then these SNPs were treated as the same QTL, and the SNP with the smallest P-value was taken as lead SNP. The contribution rate of this SNP was the contribution rate of the QTL. The functions and annotations of genes within the QTL intervals were investigated using the phytozome genome database (https://phytozome-next.jgi.doe.gov/).

### 2.4 RNA-seq statistics

Based on previous identifications in the laboratory, Lijiangxintuanheigu (LTH) and Dongnong 415 (DN415) were chosen as the susceptible and resistant varieties, respectively. When LTH and DN415 rice grew to the third to fourth leaf stage, they were subjected to spray inoculation. Before inoculation (0 dpi) and on the third day after inoculation (3 dpi), three biological replicates of detached leaf samples were collected for each variety, resulting in 6 samples per variety and a total of 12 samples. Total RNA extraction from the 12 samples was carried out using the TransZol Up RNA Kit (Invitrogen, Carlsbad, CA, USA). The RNA amount and purity of each sample was quantified using NanoDrop ND-1000 (NanoDrop, Wilmington, DE, USA). The RNA integrity was assessed by Bioanalyzer 2100 (Agilent, CA, USA) with RIN number >7.0, and confirmed by electrophoresis with denaturing agarose gel. Poly (A) RNA is purified from 1μg total RNA using Dynabeads Oligo (dT)25-61005 (Thermo Fisher, CA, USA) using two rounds of purification. Then the poly(A) RNA was fragmented into small pieces using Magnesium RNA Fragmentation Module (NEB, cat.e6150, USA) under 94°C 5-7min. Then the cleaved RNA fragments were reverse-transcribed to create the cDNA by SuperScript™ II Reverse Transcriptase (Invitrogen, cat. 1896649, USA), which were next used to synthesise U-labeled second-stranded DNAs with E. coli DNA polymerase I (NEB, cat.m0209, USA), RNase H (NEB, cat.m0297, USA) and dUTP Solution (Thermo Fisher, cat.R0133, USA). An A-base is then added to the blunt ends of each strand, preparing them for ligation to the indexed adapters. Each adapter contains a T-base overhang for ligating the adapter to the A-tailed fragmented DNA. Single- or dual-index adapters are ligated to the fragments, and size selection was performed with AMPureXP beads. After the heat-labile UDG enzyme (NEB, cat.m0280, USA) treatment of the U-labeled second-stranded DNAs, the ligated products are amplified with PCR by the following conditions: initial denaturation at 95°C for 3 min; 8 cycles of denaturation at 98°C for 15 sec, annealing at 60°C for 15 sec, and extension at 72°C for 30 sec; and then final extension at 72°C for 5 min. The average insert size for the final cDNA library was 300 ± 50 bp. At last, we performed the 2×150bp paired-end sequencing (PE150) on an Illumina Novaseq™ 6000 (LC-Bio Technology CO., Ltd., Hangzhou, China) following the vendor’s recommended protocol. HISAT was utilized to construct the index and map clean reads to the reference genome. FPKM values of transcripts were calculated using StringTie and ballgown. Differential expression analysis was conducted using edgeR, with significance defined as *P* < 0.05 and |log_2_FC| > 1 (Robinson et al., 2010).

### 2.5 Haplotype analysis

We retrieved the positional information of genes within the QTL intervals identified by GWAS analysis from the Phytozome13 database (https://phytozome-next.jgi.doe.gov/). Non-synonymous mutations from the exon regions of genes were extracted using the RiceSNP Seek Database (https://snp-seek.irri.org/) and combined with SNPs located within a 2kb range upstream of the start codon for haplotype analysis.

### 2.6 qRT-PCR

Total RNA was extracted from the samples using the TranZol Up RNA kit from TransGen Biotech following the manufacturer’s instructions. Subsequently, cDNA synthesis was performed using the HiFiScript cDNA Synthesis Kit from Cwbio according to the provided protocol. qRT-PCR was conducted using the BlazeTaq SYBR Green qPCR Mix 2.0 detection reagent on the Roche LightCycler96 instrument. The reaction program included a 2-minute denaturation at 94°C, followed by 40 cycles of 94°C for 15 seconds, 60°C for 35 seconds, and 72°C for 30 seconds. Fluorescence signals were collected during the 72°C extension step. To ensure the reliability of the results, each sample was run in three technical replicates. Relative quantification was used to calculate the expression levels of the genes. After confirming that the expression level of the reference gene *(OsActin1*) remained stable across all treatment groups, the threshold cycle (CT) values for each gene were recorded, and the relative expression levels of the target genes in the samples were calculated based on the CT values. Primer design was conducted using Primer 5.0 software, with the rice *OsActin1* gene utilized as the internal reference gene for normalization (**Supplementary Table S2**).

### 2.7 Bioinformatics analysis of OsLB2.2

To initially explore some of the functions of the *OsLB2.2* gene related to disease resistance that was screened out, we conducted an analysis of this gene. The protein sequence of OsLB2.2 was uploaded to the NCBI website for analysis. The tetratricopeptide repeat (TPR) domain of OsLB2.2 protein was extracted and compared with protein sequences from rice, Arabidopsis thaliana, and maize obtained from the Ensembl database (https://www.ensembl.org/index.html), which has high data integration. Subsequently, the TPR domain of OsLB2.2 was designated as the reference sequence for further analysis. Using Blastp (version 2.15.0) with a significance threshold of 1e-5, direct homologous proteins of rice, Arabidopsis thaliana, and maize were identified. Multiple sequence comparisons between the homologous proteins and the amino acid sequences of OsLB2.2 were conducted using Muscle (version 5.1). The evolutionary tree was constructed utilizing the Neighbor-Joining (NJ) method in Mega (version 11.0.13) and further refined aesthetically using Evolview v2 (https://evolgenius.info).

### 2.8 *OsLB2.2* mutant plants

The homozygous T_1_ generation mutant seeds with Zhonghua11 (ZH11) background were obtained from BIOGLE GENETECH company (http://www.biogle.cn/) by CRISPR/Cas9 method in April 2023. The T1 generation seeds were planted in Harbin of China, and the homozygous T_2_ generation seeds were obtained in October 2023 for subsequent disease resistance assessments.

### 2.9 Development and validation of specific KASP markers

To further clarify the role of *OsLB2.2* in the disease resistance of japonica rice, we sequenced and analyzed the 107 rice varieties. The promoter region and gene sequence of *OsLB2.2* in 107 rice germplasm resources were analyzed by Sanger method (**Supplementary Table S3**). According to the base sequences corresponding to SNPs and INDELs, KASP primers were designed using the Primer3plus software, including two specific primers and one universal primer. Each specific KASP primer was respectively linked to the FAM (5’-GAAGGTGACCAAGTTCATGCT-3’) and HEX (5’-GAAGGTCGGAGTCAACGGATT-3’) adapter sequences. The primer sequences are as shown in (**Supplementary Table S4**). KASP genotyping was carried out using the PCR part of the GeneSmart 2000 instrument from Hanchuang Guangyi Company.

## 3 Results

### 3.1 Phenotypic data analysis

**Figure 1:**
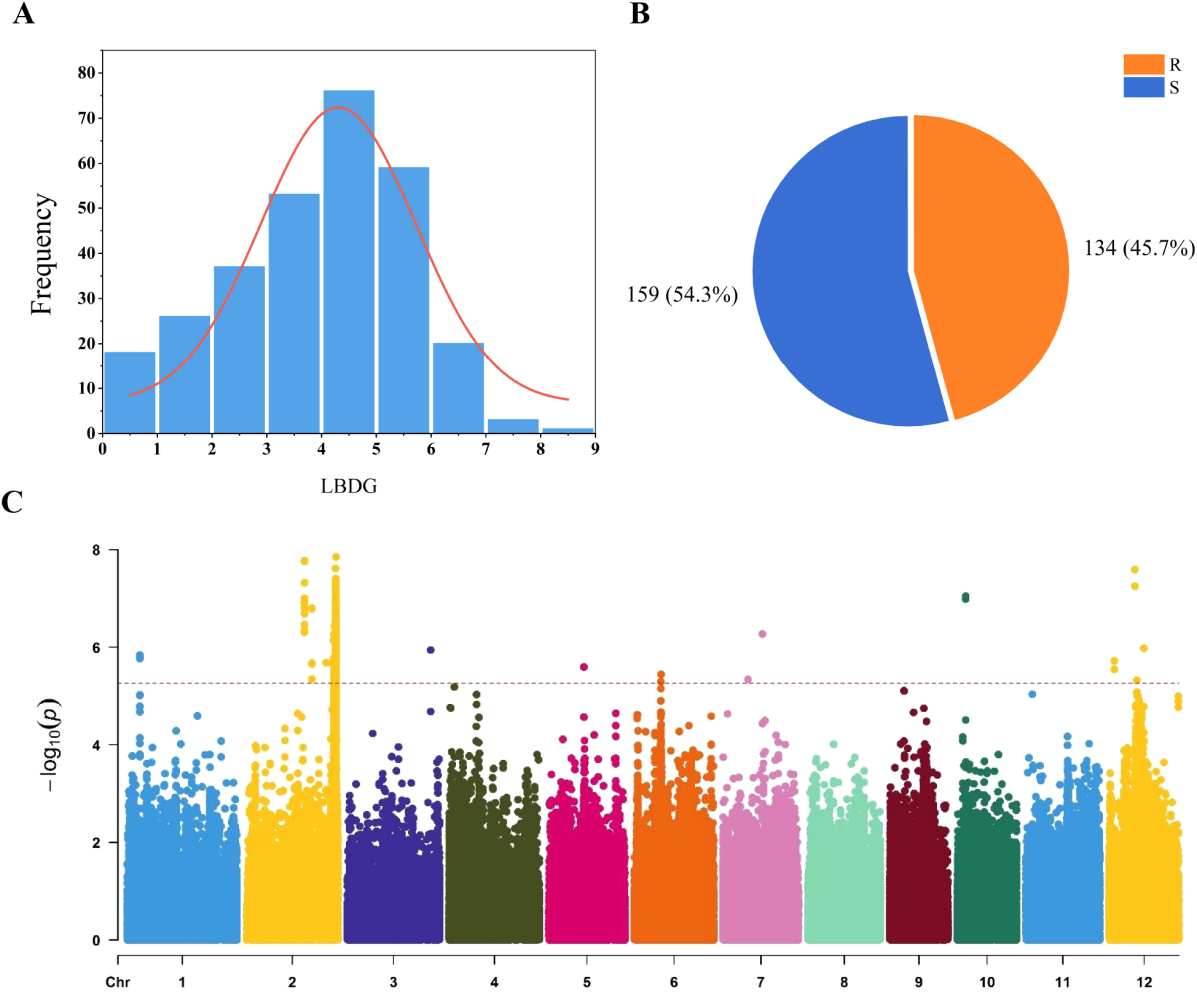
Frequency distribution of seedling blast classes and resistance classification, along with the Manhattan plot of seedling leaf blast disease grade. **(A)** Distribution of 295 rice disease grades; LBDG: leaf blast disease grade; **(B)** R: resistant (R < 4); S: susceptible (4 ≤ S); **(C)** Manhattan plot of seedling leaf blast disease grade.

0In the disease grade evaluation of 295 rice varieties, disease classes 4 and 5 exhibited the highest frequency, whereas classes 7, 8, and 9 had the lowest representation (**Figure 1A**). This distribution aligns closely with a normal distribution pattern, indicating suitability for subsequent GWAS analysis. Following the inoculation and characterization of all rice materials, the varieties were classified into two disease incidence levels: 134 resistant (**Supplementary Table S5**), 159 susceptible varieties (**Figure 1B**). The mean, standard deviation and coefficient of variation of blast grade were 3.83, 1.65 and 0.43, respectively.

### 3.2 Identification of the candidate region by GWAS

A GWAS identified 11 leading SNPs associated with rice blast resistance using a mixed linear model (MLM) from a set of 788,396 high-quality SNPs (**Figure 1C**). These 11 rice blast resistant loci were identified. Their phenotypic contribution ranged from 8.12% to 13.23%. The intervals containing the 11 leading SNPs were designated as *qLB1*, *qLB2.1*, *qLB2.2*, *qLB3*, *qLB5*, *qLB6*, *qLB7.1*, *qLB7.2*, *qLB10*, *qLB12.1*, and *qLB12.2*. A total of 233 genes were identified within the intervals of these 11 QTLs (**Table 1**). Among them, 77 exhibited distinct haplotype patterns, with 60 genes displaying 2 haplotypes and 17 genes exhibiting 3 haplotypes. By combining the seedling rice blast phenotype data of 295 accessions, different haplotypes were associated with phenotypic variation of 40 genes (**Supplementary Table S6**). These 40 genes are distributed across four intervals, with 20 genes in *qLB2.2*, 4 genes in *qLB6*, 1 gene in *qLB7.2*, and 15 genes in *qLB12.2*.

**Table 1:** QTLs associated with leaf blast disease grade at seedling stage identified by GWAS.

QTL	Chr.	Physical region	Lead SNPs position	P value	R2(%)	Gene number
*qLB1*	1	4,870,046-5,088,046	4,979,046	1.58E-06	8.28	26
*qLB2.1*	2	22,723,191-22,941,191	22,832,191	1.70E-08	11.57	25
*qLB2.2*	2	35,118,548-35,336,548	35,227,548	1.41E-08	13.23	37
*qLB3*	3	32,698,241-32,916,241	32,807,241	1.15E-06	8.48	37
*qLB5*	5	13,735,512-13,953,512	13,844,512	2.54E-06	9.26	17
*qLB6*	6	10,464,290-10,682,290	10,573,290	3.59E-06	9.54	15
*qLB7.1*	7	9,656,799-9,874,799	9,765,799	4.55E-06	10.37	6
*qLB7.2*	7	15,363,508-15,581,508	15,472,508	5.35E-07	12.43	18
*qLB10*	10	3,107,785-3,325,785	3,216,785	9.05E-08	13.23	2
*qLB12.1*	12	1,988,091-2,206,091	2,097,091	1.90E-06	8.12	24
*qLB12.2*	12	9,982,200-10,200,200	10,091,200	2.56E-08	12.45	26

R^2^: Phenotypic variance explained.

### 3.3 Candidate genes analysis

**Figure 2:**
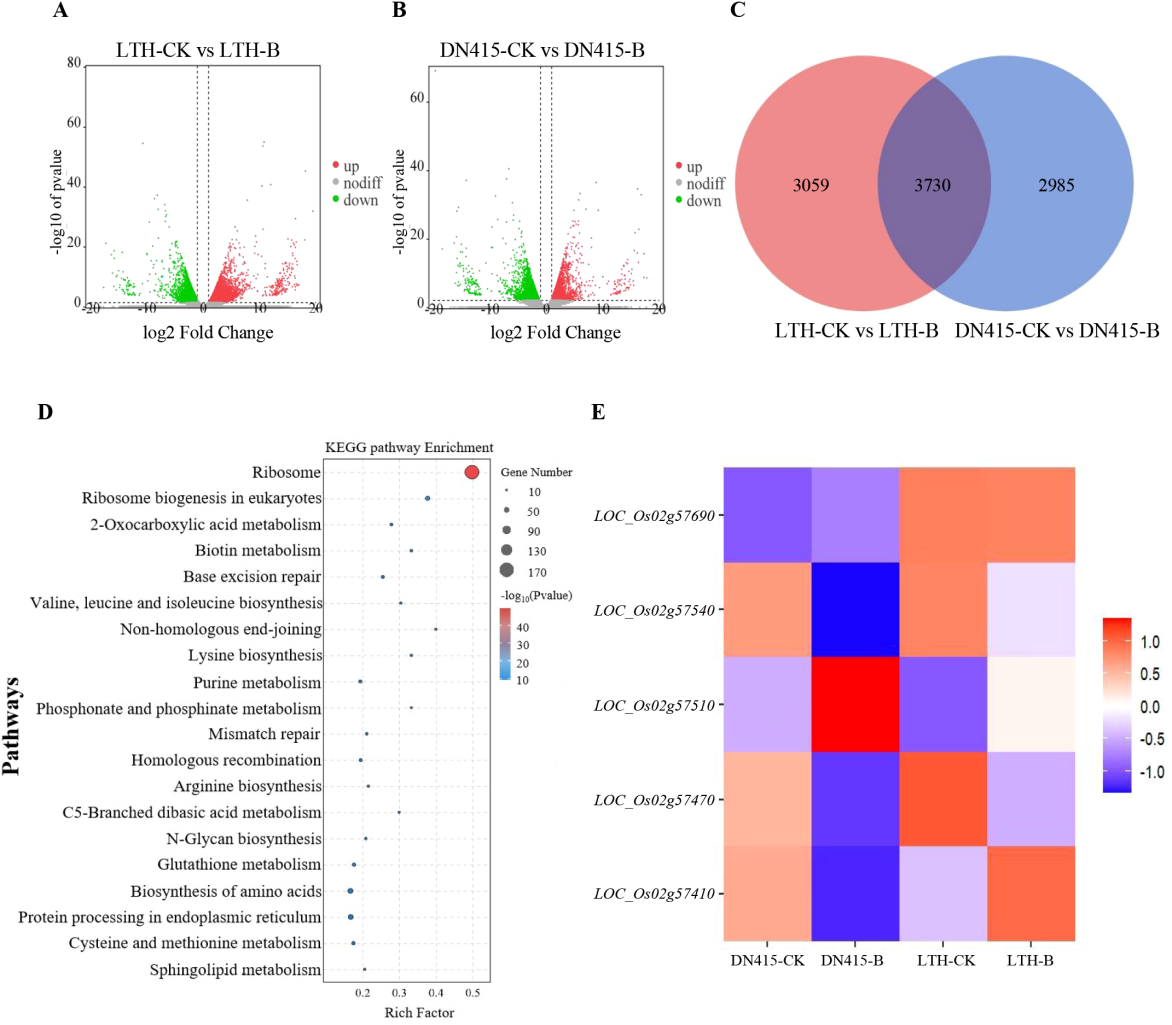
Transcriptome data analysis. **(A, B)** The volcano plots of two comparative groups. Red (Up regulated) and green (down regulated) dots indicated that the genes have signifcant expression diference, while the blue dots represent genes with no signifcant diferences. LTH-CK: The samples of Lijiangxintuanheigu treated under the condition of no inoculation treatment. LTH-B: The samples of Lijiangxintuanheigu treated under inoculation treatment. DN415-CK: The samples of Dongnong 415 treated under the condition of no inoculation treatment. DN415-B: The samples of Dongnong 415 treated under inoculation treatment. **(C)** Venn diagram of DEGs between two comparative groups. **(D)** Enrichment of biological pathways of resistance gene sets. **(E)** Gene expression heat maps of 5 candidate genes.

For further mined candidate genes, we combined previously determined RNA-seq data of the resistant variety DN415 and the susceptible variety LTH under uninoculated and inoculated conditions (**Supplementary Table S7**). As shown in **Figures 2A, B**, we took |log2FC| ≥ 1 as the threshold (*P* < 0.05) and found 6,789 differentially expressed genes (DEGs) in LTH, among which 3,596 genes were up-regulated and 3,193 genes were down-regulated. In DN415, among the 6,715 DEGs found, the numbers of up-regulated and down-regulated differentially expressed genes were 2,397 and 4,318 respectively. A total of 3730 DEGs were commonly identified in both varieties(**Figure 2C**). Genes that are specifically expressed in the resistant rice variety, along with those exhibiting a significantly higher fold change in the highly resistant variety compared to the susceptible variety ([|log2 (fc) (DN415/LTH)| ≥ 2]), collectively form a set of resistance genes candidates, totaling 3257 DEGs. To investigate the biological functions and pathways associated with the resistance gene set, a functional enrichment analysis was performed. KEGG enrichment analysis highlighted pathways such as Ribosome, Ribosome biogenesis in eukaryotes, Valine, leucine and isoleucine biosynthesis, Base excision repair, and 2-Oxocarboxylic acid metabolism as significantly enriched (*P* < 0.05), with the Ribosome pathway showing the highest degree of enrichment (**Figure 2D**). Integration of GWAS and RNA-seq revealed that 5 out of the 40 genes exhibiting haplotype variations within the QTL interval were differentially expressed in the set of disease resistance genes (**Supplementary Table S8**; **Figure 2E**). Among these, 5 differentially expressed genes, namely *LOC_Os02g57410*, *LOC_Os02g57470*, *LOC_Os02g57510*, *LOC_Os02g57540*, and *LOC_Os02g57690*, were identified as candidate genes for seedling rice blast resistance.

### 3.4 qRT-PCR analysis

**Figure 3:**
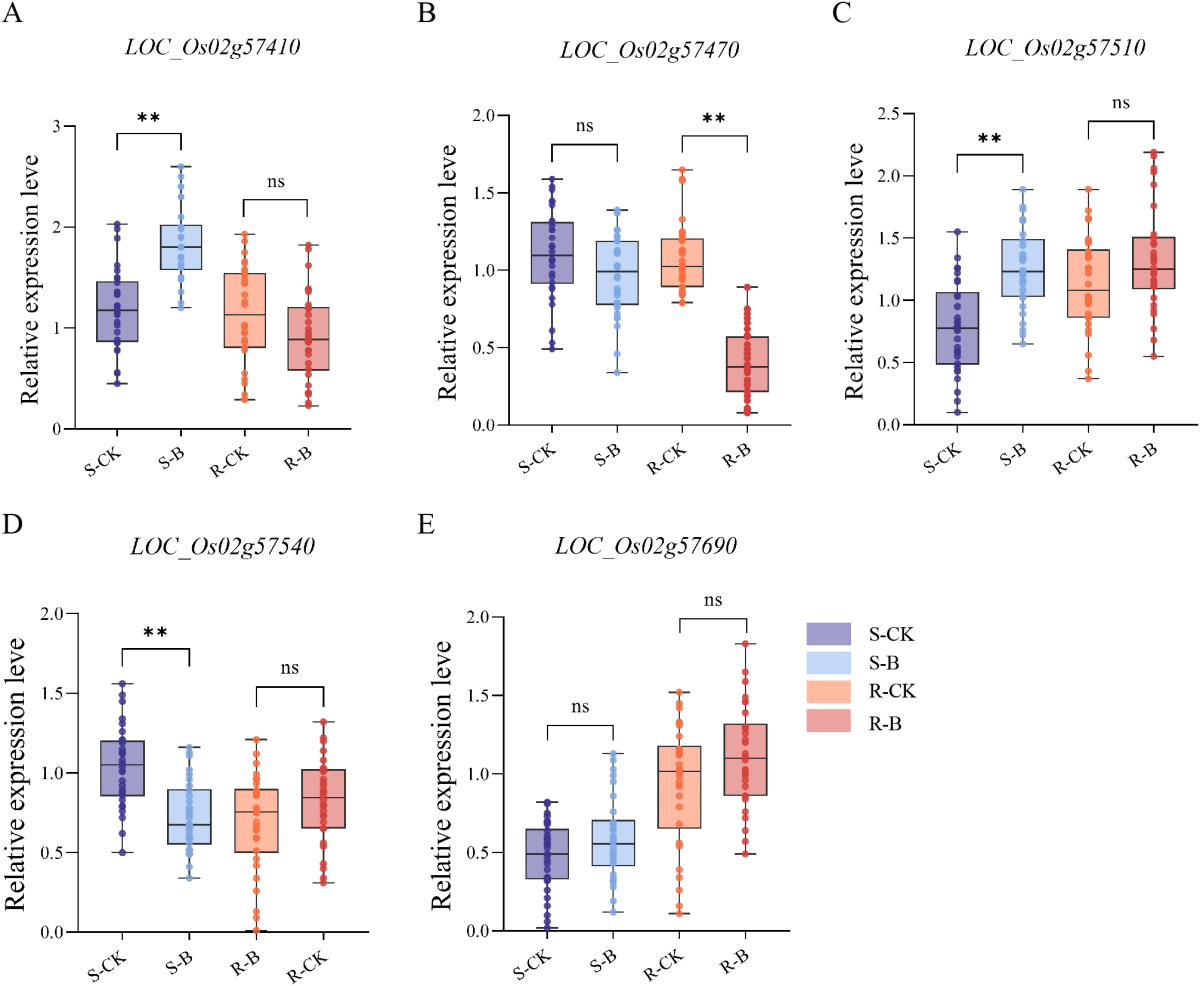
Changes in relative expression of 5 candidate genes in resistant and susceptible varieties. **(A–E)** The expression of *LOC_Os02g57410, LOC_Os02g57470, LOC_Os02g57510, LOC_Os02g5754*0, and *LOC_Os02g57690* genes under inoculation conditions were analyzed. S-CK: The samples of 10 susceptible varieties treated under the condition of no inoculation treatment. S-B: The samples of 10 susceptible varieties treated under inoculation treatment. R-CK: The samples of 10 resistant varieties treated under the condition of no inoculation treatment. R-B: The samples of 10 resistant varieties treated under inoculation treatment. Asterisks indicate statistical significance (**P ≤ 0.01, t test).

To further elucidate the key genes associated with seedling blast resistance in rice, 10 susceptible and 10 resistant varieties were selected from 295 germplasm resources. Inoculation treatments were conducted, and the relative expression levels of the 5 identified genes were assessed in the 20 varieties under uninoculated and inoculated conditions. There was no significant difference in the relative expression of *LOC_Os02g57690* in susceptible or highly resistant varieties under uninoculated and inoculated conditions. The *LOC_Os02g57410*, *LOC_Os02g57510*, and *LOC_Os02g5754*0 were significant differences expression in the susceptible varieties under uninoculated and inoculated conditions. The *LOC_Os02g57470* were significant differences expression in highly resistant rice varieties under uninoculated and inoculated conditions, suggesting its potential involvement in regulating the blast resistance of rice. Subsequently, *LOC_Os02g57470* was designated as *OsLB2.2*. (**Figure 3**).

### 3.5 Functional validation of *OsLB2.2* gene

**Figure 4:**
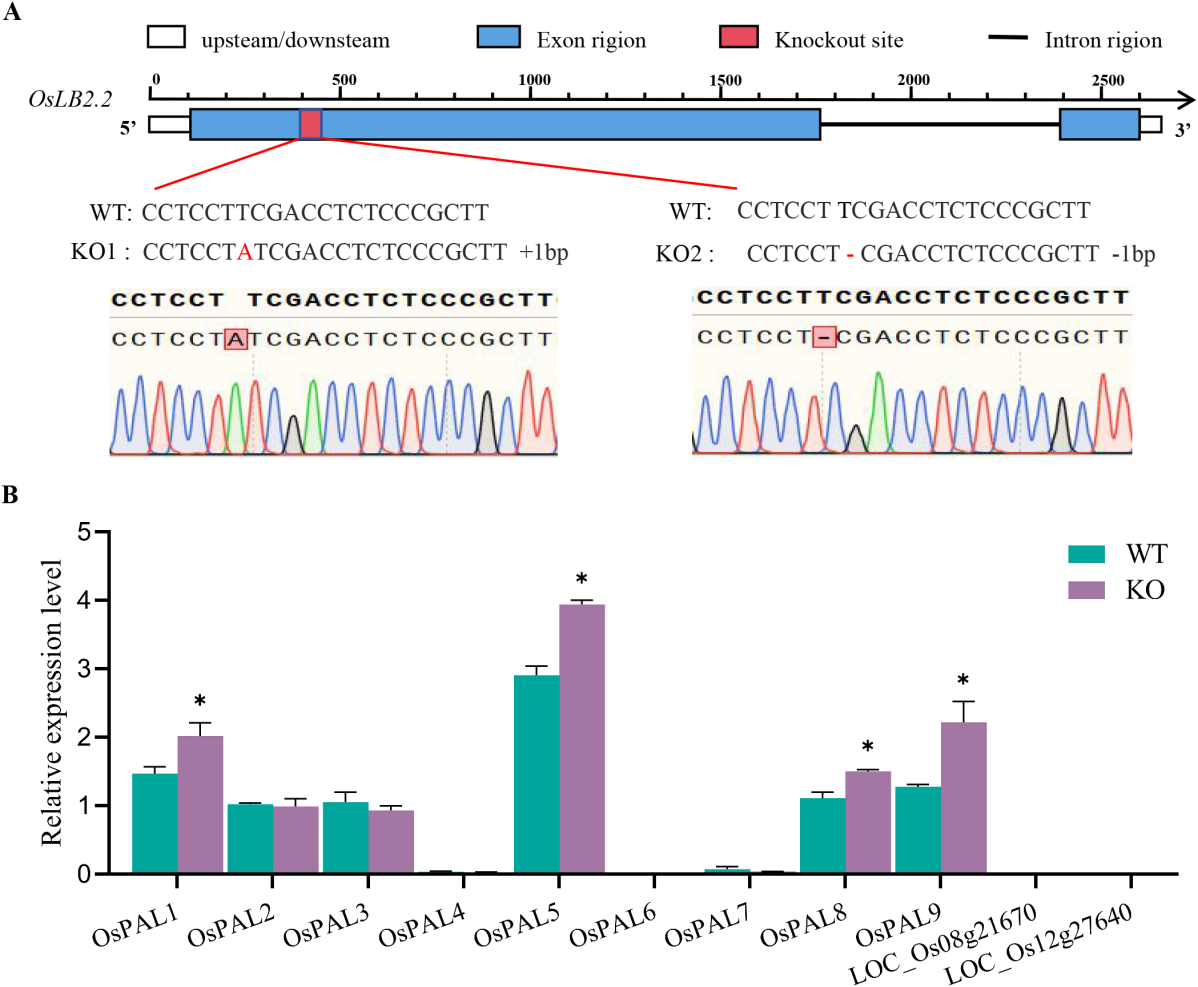
The target sequence of *OsLB2.2* and relative expression levels of *PAL* family genes. **(A)** DNA sequences of *OsLB2.2* in ZH11 (WT) and *oslb2.2* knockout plants (KO1, KO2); **(B)** Relative expression levels of PAL family genes of WT and *oslb2.2* knockout plants. Asterisks indicate statistical significance (*P ≤ 0.05, t test).

To clarify the function of *OsLB2.2* in rice blast resistance, two mutants *oslb2.2* (KO1, KO2) with the background of Zhonghua 11 (WT) were constructed by gene editing technology (**Figure 4A**). Disease investigations were carried out on the mutants and the WT at 7 d after inoculation. Compared with the WT plants, the mutants plants lesion area and leaf blast grade were significantly reduced, indicating that *oslb2.2* acts as a negative regulator of rice blast resistance. Through further bioinformatics analysis, the *OsLB2.2* protein was found to contain four TPR domains (**Supplementary Figure S1A**), and 24 homologous genes in rice were obtained (**Supplementary Figure S1B**). TPR-containing proteins have been reported to regulate *PAL* genes mRNA metabolism. Loss of the TPR proteins leads to accumulation of *PAL* genes mRNAs in the mutant which in turn enhanced the disease resistance of the plant (Zhou et al., 2018). In this study, the gene expression levels of the *PAL* gene family were measured in the *oslb2.2* mutant and WT (**Supplementary Figure S2**), among them, the expressions of 4 genes were up-regulat ed in *oslb2.2* mutant, indicated that OsLB2.2 may mediate the expression of *PALs* and participate in the regulation of rice blast resistance (**Figure 4B**).

### 3.6 Natural variation analysis in *OsLB2.2*

**Figure 5:**
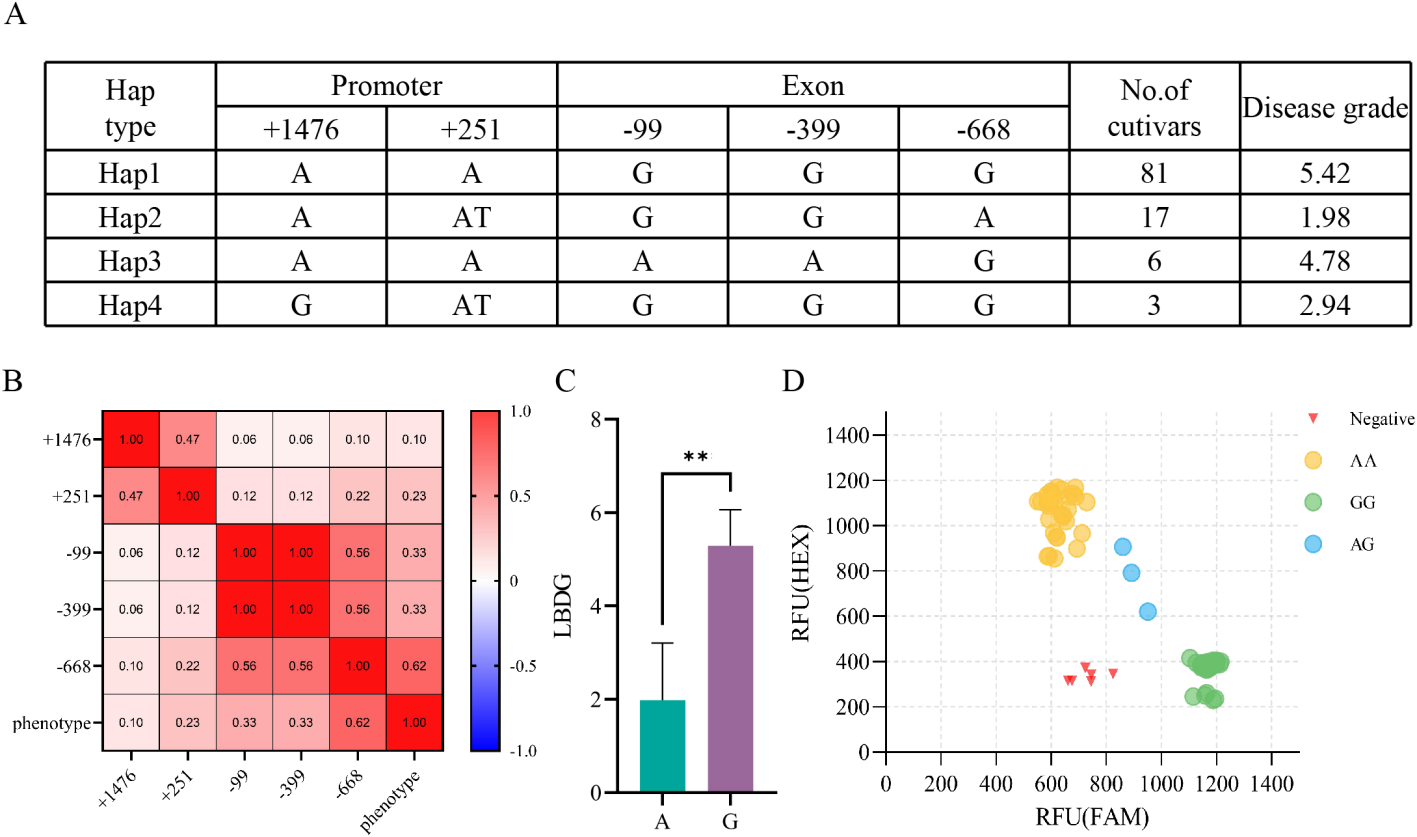
Natural variation analysis in *OsLB2.2.* **(A)** Haplotype analysis of the *OsLB2.2* gene region from 107 temperate japonica. **(B)** Analysis of correlation between variation sites and phenotypes. **(C)** Unit point analysis. **(D)** Typing results of KASP marker on 107 populations **P≤ 0.01, t test.

To further clarify the role of *OsLB2.2* in the disease resistance of japonica rice, we sequenced and analyzed the *OsLB2.2* promoter and gene sequence in 107 rice varieties. There were 4 SNPs and 1 INDEL in the promoter region and exon region of *OsLB2.2*. Among them, Hap2 with average disease grades of 1.98 respectively under inoculation conditions. In contrast, the average disease grades with Hap1, Hap3 and Hap4 genotypes were 5.42, 4.78 and 2.94 respectively (**Figure 5A**). By further analyzing the correlation between the variant sites and the phenotype, it was found that the SNP (-668) site was significantly correlated with the phenotype (**Figures 5B, C**), suggesting that this SNP might be associated with the rice blast resistance mediated by OsLB2.2. To promote the development of rice varieties resistant to rice blast using this gene, we designed a KASP marker for the SNP site and genotyped these 107 varieties (**Figure 5D**). The genotyping results showed that the KASP markers could accurately distinguish the genotypes of *OsLB2.2* gene in different rice germplasms, and could be applied to the subsequent molecular improvement of rice blast.

## 4 Discussion

Blast is one of the main diseases of rice, which seriously affects the rice yield and quality (Guo et al., 2018). The development of resistant rice varieties is a crucial strategy to mitigate yield losses (Fernandez and Orth, 2018). Chen et al. (2019) found that rice susceptibility to blast is more pronounced during the 1 to 4 leaf stage, emphasizing that identification beyond the 4 leaf stage may obscure the susceptibility of rice varieties. Many studies have screened a series of valuable rice blast resistance germplasm resources by rice blast identification at seedling stage (Li et al., 2019a; Krishna et al., 2020; Liang et al., 2020; Peng et al., 2021). These studies indicate that screening for resistant varieties through blast resistance assessment at the seedling stage is a more efficient approach. In this study, we identified 44 high resistant blast rice germplasm resources through the identification of rice blast resistance of 295 rice varieties at seedling stage. Due to the dynamic changes of rice blast flora, the service life of rice blast resistant varieties is short (Chen et al., 2001; Nguyet et al., 2020). It is an effective method to improve the service life of rice varieties by introducing rice germplasm resources resistant to rice blast in different regions and using them for shuttle breeding. In this study, the high resistance blast rice varieties identified provide valuable germplasm resources for rice blast resistance improvement breeding.

In recent years, GWAS has been widely used in association gene mining of major traits in many crops. In the aspect of rice blast resistance gene mining, *Pb2*, *Pb3*, *Pb4*, *PIkx* and *PiPR1* rice blast resistance genes were identified by GWAS (Li et al., 2019a; Liu et al., 2020; Yu et al., 2022; Ma et al., 2022; Fan et al., 2024). In this study, we identified 11 QTLs conferring rice blast resistance by GWAS, encompassing 233 genes in these regions. By analyzing the haplotypes of different rice varieties and the corresponding phenotypic data, the haplotypes associated with specific phenotypes can be identified (Bhat et al., 2021). The genomic regions where these associated haplotypes are located often contain candidate genes that affect the phenotype, thereby quickly locating the regions in the genome related to the target phenotype and narrowing the search range of candidate genes (Li et al., 2022). In this study, 40 genes within the 11 QTL intervals obtained through haplotype analysis. RNA-Seq has been more and more widely used to identify disease resistance pathways or resistance genes with the development of next‐generation sequencing. With transcriptomic analysis, Yang et al. (2021) determined the key genes for immune response of rice inoculated with rice blast, which included *OsMT1a*, *OsMT1b* and *Perox4*. In this study, identification of disease resistance gene with 3,257 DEGs was made possible using transcriptomic analysis. The results of RNA-seq and qRT-PCR analysis showed that *OsLB2.2* may be involved in the regulation of rice blast resistance.

In this study, structural analysis of *OsLB2.2* showed the gene contained 4 tetratricopeptide repeat (TPR) domains. In maize, a putative TPR protein encoded by *ZmTDM1* gene functions as a regulator of the meiotic cell cycle and was required for exit from meiosis II (Zhang et al., 2024). The TPR gene *Mai1*, which is also expressed in tomato and encodes a protein related to M3F, interacts with M3Kα and seems to increase stimulation of MAPK, also resulting in immune related programmed cell death (Roberts et al., 2019). In rice, *Bsr-k1* encodes a TPR protein with RNA binding activity, and the disease resistance of *bsr-k1* mutant was significantly enhanced compared with WT plant (Zhou et al., 2018). In this study, knockout of *OsLB2.2* also significantly enhanced the blast resistance of rice, indicating that *OsLB2.2* can negatively regulate rice blast resistance and *OsLB2.2* gene editing can rapidly improve rice blast resistance. Previously study result shown that TPR proteins bind the mRNA of the *PAL* gene family encoding enzymes involved in the regulation of phenylalanine metabolism. These mRNAs are degraded when TPR proteins bind, thus decreasing lignin synthesis and the immune response (Younas et al., 2024). In this study, the expression of *PAL* family genes was analysed in both WT and *oslb2.2* mutant plant, found that the expression of four genes *OsPAL9* (Yan et al., 2015), *OsPAL8* (He et al., 2020), *OsPAL5*, and *OsPAL1* (Wang et al., 2022b) were significantly increased in the *oslb2.2* mutant plants, suggesting that binding of *OsLB2.2* might also improve the rice blast resistance via regulating the *PALs*.

Natural variation is the raw material of speciation as well as the means by which organisms evolve better adaptation to the environment, through natural selection. Variation in the wild soybean *GsERD15B* promoter has been demonstrated to enhance salt tolerance, and provides important theoretical foundations in soybean breeding and use in saline-alkaline soil agricultural production (Jin et al., 2021). Among the known resistance genes, *Pi9* locus has the most homologous genes in series, among which homologous genes such as *Pi9*, *Pi2* and *PigmR* have good rice blast resistance in various rice planting areas and have been widely used. Yang et al. (2023) developed specific molecular markers for six resistance genes, such as *Pi2*, *Piz-t, Pi9*, *Pi9-type5*, *PigmR* and *Pid4*, and applied them to rice blast resistance improvement breeding. In this study, we identified the *OsLB2.2* gene haplotype, of which Hap2 were the dominant haplotypes. The SNP site with the strongest specificity was further obtained, and the *OsLB2.2* specific molecular marker was developed and verified. These result that will allow for genetic improvement of rice blast resistance.

## Data Availability

The datasets presented in this study can be found in online repositories. The names of the repository/repositories and accession number(s) can be found below: https://www.ncbi.nlm.nih.gov/, PRJNA1123870.

## Supplementary material

Supplementary Figure S1 Analysis of the protein structure and phylogenetic tree of OsLB2.2.

Supplementary Figure S2 Phylogenetic tree of the PAL gene family.
