# Supplementary figures and images for: OsLB2.2 negatively regulates rice disease resistance at seedling stage in rice
Source: Front Plant Sci. 2025 Aug 11;16:1629283. doi: 10.3389/fpls.2025.1629283 (PMC12375565; doi:10.3389/fpls.2025.1629283)

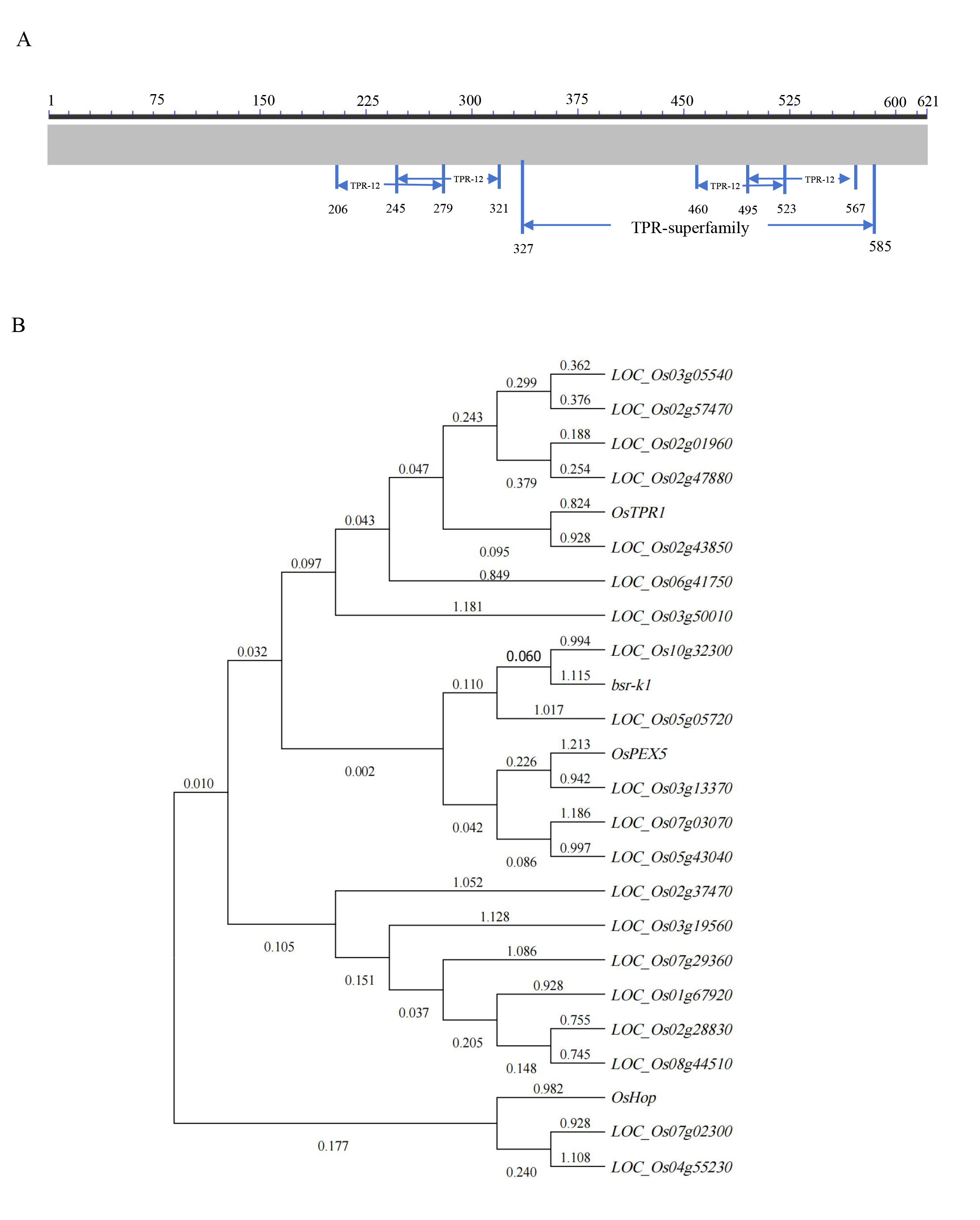

Supplement: Supplementary Figure S1 — Analysis of the protein structure and phylogenetic tree of OsLB2.2. [file Image1.tif]

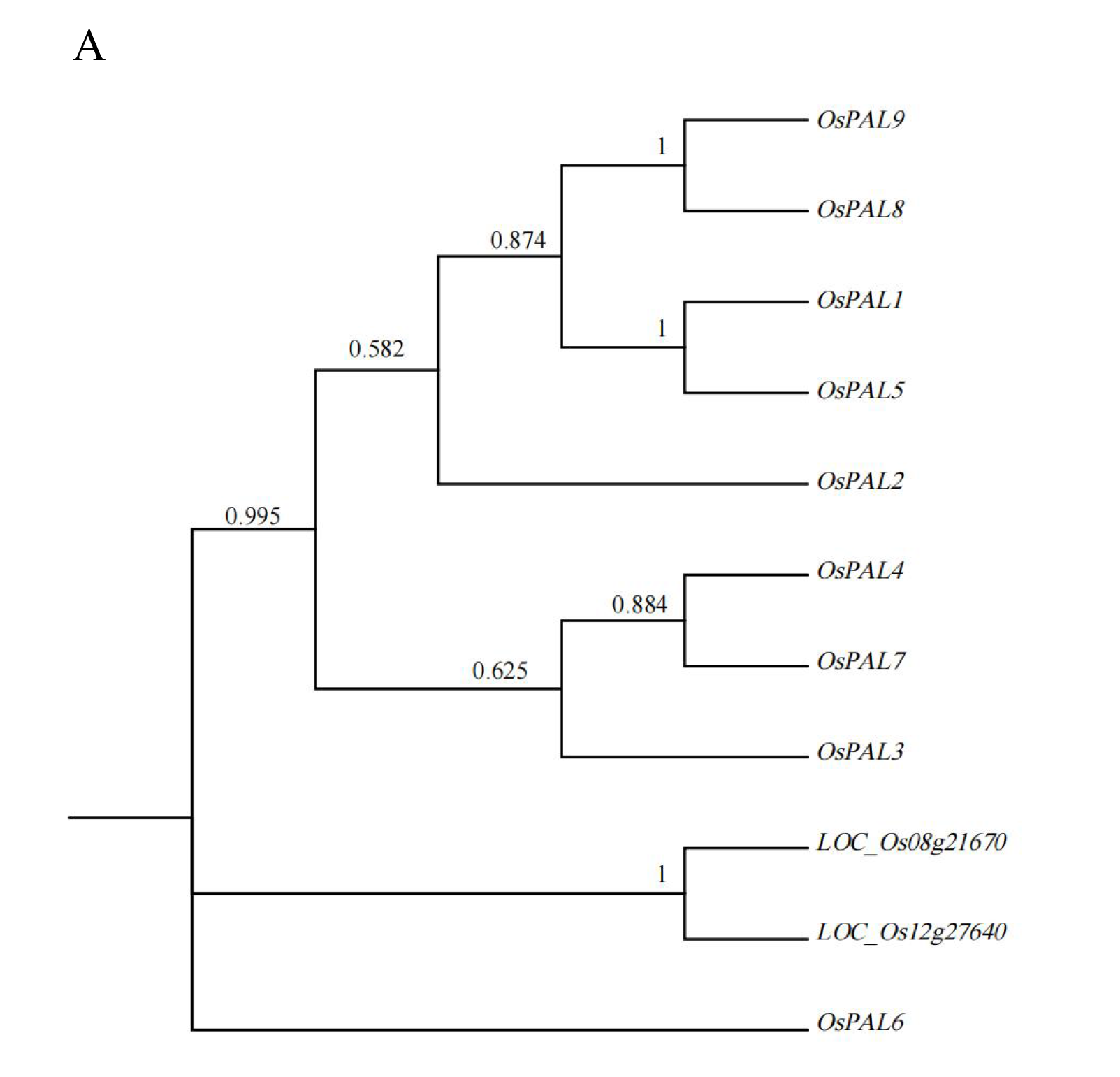

Supplement: Supplementary Figure S2 — Phylogenetic tree of the PAL gene family. [file Image2.tif]
